# Extremes of weight centile are associated with increased risk of mortality in pediatric intensive care

**DOI:** 10.1186/cc10127

**Published:** 2011-03-31

**Authors:** Andrew Numa, John McAweeney, Gary Williams, John Awad, Hari Ravindranathan

**Affiliations:** 1 Intensive Care Unit, Sydney Children's Hospital, High Street, Randwick 2031, Australia; 2 University of New South Wales, Anzac Parade, Kensington 2033, Australia

## Abstract

**Introduction:**

Although numerous studies have linked extremes of weight with poor outcome in adult intensive care patients, the effect of weight on intensive care outcome has not previously been reported in the pediatric population. The aim of this study was to investigate the relationship between admission weight centile and risk-adjusted mortality in pediatric intensive care patients.

**Methods:**

Data were collected on 6337 consecutively admitted patients over an 8.5 year period in a 15 bed pediatric intensive care unit (ICU) located in a university-affiliated tertiary referral children's hospital. A weight centile variable was entered into a multivariate logistic regression model that included all other pediatric index of mortality (PIM-2) variables, in order to determine whether weight centile was an independent risk factor for mortality.

**Results:**

Weight centile was associated with mortality in both univariate and multivariate analysis, with the lowest mortality being associated with weights on the 75^th ^centile and increasing symmetrically around this nadir. A transformed weight centile variable (absolute value of weight centile-75) was independently associated with mortality (odds ratio 1.02, *P *= 0.000) when entered into a multivariate logistic regression model that included the PIM-2 variables.

**Conclusions:**

In this single-center cohort, weight centile was an independent risk factor for mortality in the ICU, with mortality increasing for patients at either end of the weight spectrum. These observations suggest that the accuracy of mortality prediction algorithms may be improved by inclusion of weight centile in the models. A prospective multicenter study should be undertaken to confirm our findings.

## Introduction

Nutritional status has significant effects on morbidity and mortality in the general population. Obesity is well recognized as a risk factor for many disorders of adult life, including diabetes, hypertension, coronary vascular disease, osteoarthritis, depression, and some malignancies, and significantly increases all-cause mortality [1-4]. At the other end of the spectrum, nutritional deficiency is a major contributor to infant and child mortality throughout the world [5,6], and body mass index (BMI) of less than 18.5 kg/m^2 ^has been associated with minor increases in all-cause mortality in adults [1].

Studies in adult intensive care patients have demonstrated a variable relationship between BMI and mortality. A number of studies have failed to demonstrate any impact of body mass on intensive care outcome [7-11], whereas others have demonstrated an association between obesity and increased risk-adjusted mortality [12-14]. A considerable body of evidence suggests that underweight adult intensive care patients are at the greatest risk for mortality, and risk-adjusted odds ratios (ORs) for death of 1.16 to 1.63 compared with patients of normal weight have been reported [15-17]. No studies have addressed the impact of body weight on outcome in the pediatric intensive care unit (PICU), although Larsen and colleagues [18] noted that low weight (but not age) was an independent risk factor for mortality in children undergoing cardiac surgery. We undertook this study to explore the relationship between weight centile and risk-adjusted mortality in PICU patients.

## Materials and methods

Sydney Children's Hospital is a university-affiliated pediatric tertiary referral center with all medical and surgical subspecialties represented. The ICU annually admits approximately 850 patients who range in age from birth to 16 years and is one of three tertiary pediatric centers in the state of New South Wales, serving a total population of approximately 6.77 million, including 1.32 million children who are 14 years old or younger. A separate neonatal ICU (NICU) on campus provides care for premature infants; however, infants born with complex surgical conditions (for example, congenital diaphragmatic hernia and structural heart disease) are generally managed in the PICU rather than the campus NICU. The ICU offers a full range of supportive therapies, including inhaled nitric oxide, high-frequency ventilation, hemofiltration, and extra-corporeal membrane oxygenation.

All patients admitted to the ICU between 1 January 2002 and 30 June 2010 were eligible for inclusion in this study. Body weight, obtained from recent health records, parental knowledge, or direct measurement, was recorded on admission to the ICU. PIM-2 (Pediatric Index of Mortality version 2) variables [19], along with body weight, were prospectively recorded in all patients. Weight-for-age *z*-scores were calculated from data from the Centers for Disease Control and Prevention [20] and converted to centiles. For preterm infants less than 2 years old at admission, corrected age was used in preference to chronological age. If corrected age was less than term, preterm growth charts were used to calculate weight centile [21].

The relationship between weight centile and mortality was explored by using Copas *p *by *x *plots [22], and statistical significance was confirmed by using the Mann-Whitney *U *test. Univariate and multivariate logistic regression was performed to explore the relationship between PIM-2 variables together with weight centile and mortality. Standardized mortality rates were calculated by using PIM-2 coefficients [19], and statistical significance was determined by Poisson analysis; binomial proportions were compared by using standard equations [23]. Comparison of areas under receiver operating characteristic (ROC) curves was carried out by using likelihood ratio testing [24]. Statistics were analyzed by using SPSS 18.0.2 (IBM Corporation, Armonk, NY, USA) and GraphPad Prism 5.0 (GraphPad Software, Inc., La Jolla, CA, USA). The study was approved by the ethics committee of the South Eastern Sydney and Illawarra Health Service, and informed consent was not required for this analysis of data, which are routinely collected on all ICU patients.

## Results

Six thousand three hundred thirty-seven patients were admitted to the ICU during the study period. Of these, 16 had no weight recorded and 5 had a history of prematurity with no gestational age recorded; these patients (*n *= 21) were excluded from further analysis. There were 203 deaths in the 6,316 remaining patients, a mortality of 3.2%. The PIM-2-predicted mortality was 226.2, giving a standardized mortality rate of 0.897, which is significantly better than predicted (*P *= 0.008).

**Figure 1:**
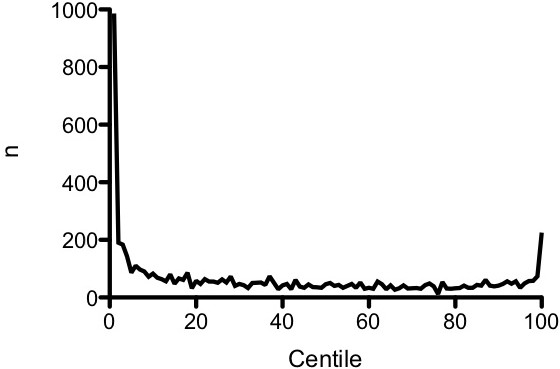
**Distribution of patients by weight centile**.

**Figure 2:**
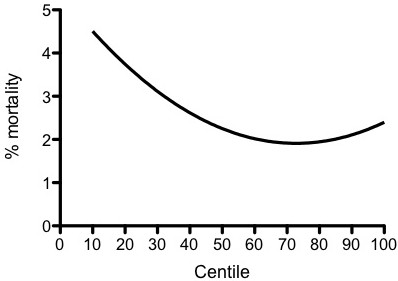
**Mortality versus weight centile**. Distance-weighted least squares plot is shown.

Patients at the extremes of the weight spectrum were over-represented numerically (Figure 1). For example, 21.5% of patients had weights not above the 3rd centile and 5.7% had weights on at least the 97th centile compared with the expected 3% in each group if the population was distributed normally (*P *< 0.001). The relationship between weight centile and mortality was symmetrical around a nadir at the 75th centile (Figure 2), and mortality at the lower end of the weight spectrum was more than double that of patients at the 75th centile nadir. Weight centile was thus transformed before inclusion in the multivariate regression by using the absolute value of weight centile minus 75 (that is, a patient with a weight on the 5th centile would have a transformed value of 70 used as the weight term in the regression, and a patient with a weight on the 85th centile would have a transformed value of 10). This transformed weight variable was then entered into a multivariate logistic regression model that included the PIM-2 variables. All PIM-2 variables, with the exception of elective admission (*P *= 0.486) and bypass (*P *= 0.069) status, were statistically significant in the model. As there was a very high correlation in our cohort between the 'elective admission' and 'recovery post-procedure' categories, with only 283 (11.8%) of 2,402 elective admissions not being admitted from the recovery room, it is not surprising that elective admission status was not an independent predictor in a model that included both variables (each was significant in univariate analysis). Similarly, only 407 patients (6.4%) were admitted following cardiopulmonary bypass, and this variable just failed to achieve statistical significance.

**Figure 3:**
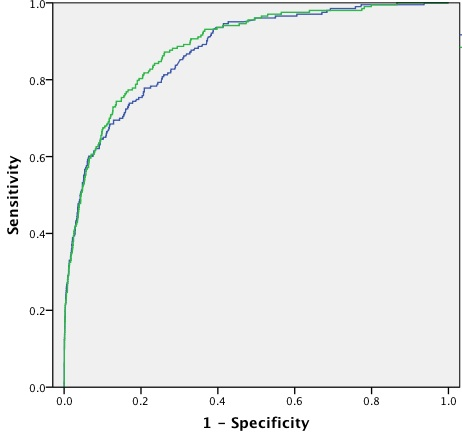
**Comparison of receiver operating characteristic curves for PIM-2 variables with (green curve) and without (blue curve) the weight variable included in the model**. PIM-2, Pediatric Index of Mortality version 2.

The transformed weight centile variable was significant in univariate and multivariate analyses (OR for death of 1.02; *P *= 0.000 in multivariate analysis). Eliminating elective status and bypass status from the regression model did not significantly change the ORs or *P *values for the other variables, including weight category (OR for the transformed weight variable in the model without elective and bypass status included = 1.02; *P *= 0.000). Exclusion of premature infants from the analysis did not substantially alter the findings (data not shown). ORs for PIM-2 variables and the weight variable are shown in Table 1. Including the weight variable in the PIM-2 model increased the area under the ROC curve from 0.876 (95% confidence interval 0.851 to 0.900) to 0.887 (0.864 to 0.909) (Figure 3). This increase in area was statistically significant (*P *= 0.0002).

**Table 1:** Odds ratios for PIM-2 variables and weight centile

	PIM-2 OR	These data OR (95% CI)	*P *value
Pupils fixed to light	21.74	51.82 (18.82 to 136.32)	0.000
High-risk diagnosis	5.38	4.87 (3.31 to 7.19)	0.000
Mechanical ventilation	3.80	2.04 (1.38 to 2.99)	0.000
Bypass	2.12	1.79 (0.94 to 3.38)	0.069
100 × FiO_2_/PaO_2_	1.34	1.35 (1.08 to 1.70)	0.006
Absolute base excess	1.11	1.05 (1.02 to 1.08)	0.000
Absolute (SBP - 120)	1.01	1.02 (1.01 to 1.03)	0.000
Absolute (weight centile - 75)	-	1.02 (1.01 to 1.02)	0.000
Elective admission	0.40	0.79 (0.39 to 1.56)	0.486
Recovery after procedure	0.36	0.17 (0.08 to 0.36)	0.000
Low-risk diagnosis	0.21	0.14 (0.06 to 0.35)	0.001
Constant	-4.88	-5.06 (-4.52 to -5.59)	0.000

CI, confidence interval; FiO_2_/PaO_2_, fraction of inspired oxygen/arterial partial pressure of oxygen; OR, odds ratio; PIM-2, Pediatric Index of Mortality version 2; SBP, systolic blood pressure.

Chromosomal or syndromal disorders or both were present in 499 of 6,317 patients (7.9%) and were more common in patients with lower weight centiles (*P *< 0.001). Thirteen point eight percent of patients with weights less than the 10th centile had a chromosomal or syndromal disorder (or both) in comparison with only 2.6% of patients with weights greater than the 90th centile. However, there was no link between mortality and presence of either a chromosomal or syndromal disorder; mortality in patients with and without such disorders was 3.2% in each group.

## Discussion

Mortality prediction scores in the PICU are important tools for benchmarking unit performance. Neither of the two most used predictive scores in pediatrics, the PIM-2 and the Pediatric Risk of Mortality (PRISM III), includes weight centile as a variable [19,25]. Weight (not weight centile) was examined during the development of PIM but was not significant on univariate testing and was removed from the model [26].

Our data demonstrate that weight is an independent risk factor for outcome, with an OR that is similar to that of systolic blood pressure (OR 1.02); that is, a one percentile change in admission weight has a mortality risk effect similar to that of a 1 mm Hg change in systolic blood pressure.

Nutritional deficiency is a major contributor to infant and child mortality throughout the world [5,6] and is directly responsible for approximately 300,000 childhood deaths per year [27]. It is entirely plausible that nutritional deficiency also increases the risk of mortality in an intensive care population. Protein-energy malnutrition has wide-ranging deleterious effects on human physiology and these effects include cardiac, renal, and hepatic function and humoral and cellular immunity [27-29]. Other authors have noted associations between nutritional deficiency and the development of multiorgan failure [30,31] and mortality risk [32,33]. When observed, excess mortality among overweight adult intensive care patients has usually been attributed to respiratory and cardiovascular dysfunction [12-14] but these are less likely to be important issues in our population. Several groups have reported an increased risk of mortality in obese children with specific disorders, including leukemia and end-stage renal disease [34-36]. Unfortunately, we did not have sufficient data to examine cause of mortality in this cohort of patients.

The value of a predictive score lies in its accuracy. The existence of variables that can substantially affect patient mortality but that are not included in widely used predictive scores makes accurate comparison of standardized mortality rates difficult. Several authors have noted poor performance of discriminatory scores when applied to non-Western populations [37-40] and it is likely that nutritional status may be at least partly responsible for this observation. Thukral and colleagues [40] noted higher standardized mortality rates (calculated by both PIM-2 and PRISM) in children who had severe malnutrition and who were admitted to an Indian PICU. Our data suggest that any PICU with a relatively large proportion of low-weight-centile patients will have an inappropriately high standardized mortality rate when current PIM or PRISM models are used. 

Analysis of our patient population revealed excessive numbers of patients with very low and very high weight centiles. Patients with weight ≤3rd centile were numerically over-represented by a factor of 7 compared with the expected number, and patients with weight ≥97th centile were over-represented by a factor of 2. Pollack and colleagues [41] noted a similarly high percentage (18%) of chronically malnourished children in a PICU population. Our data indicate that low weight centile may represent a risk factor for ICU admission and also an independent risk for mortality after admission to the ICU. Many acute and chronic illnesses are associated with weight loss, and the over-representation of low-weight patients in the ICU population is biologically plausible and not unexpected. An association between low socioeconomic class and increased risk of intensive care admission has also been reported [42] and this may also contribute to increasing the proportion of patients with low weight centile in the ICU. The apparent over-representation at the other extreme of the centile range may reflect merely the increasing incidence of obesity in the community. In 2007-2008, 11.9% of American children from 2 to 19 years of age had a BMI above the 97th centile [43], while a recent study of inpatients (excluding intensive care patients) in an Australian tertiary referral children's hospital demonstrated that 11% of hospital inpatients more than 12 months old had a BMI on at least the 97th centile when measured according to the same growth parameters used for this study [44]. The same study demonstrated that only 6% of hospitalized inpatients were underweight (as defined by a weight-for-age *z*-score of less than -2.0).

One potential weakness of our study is that we did not directly measure weight in the majority of the children admitted to the ICU but instead relied on recorded hospital admission weights (most patients admitted to the non-ICU wards have weight measured on admission), parental knowledge, and infant health records. We believe that the majority of weight measurements were accurate, and any errors arising from this methodology are likely to be random rather than systematic. Random (as opposed to systematic) errors in study populations are largely overcome by enrolling sufficient numbers of patients [45]. Thus, the presence of random error will increase the probability of a type II statistical error (failing to detect significant associations) but does not invalidate a statistically significant result; we therefore believe that our findings are likely to be correct. Furthermore, measured admission weight on arrival in the ICU will be affected by the patient's hydration status, which will commonly vary over at least a 10% (that is, ±5%) range. It has been suggested that excessive volume resuscitation in critically ill adult patients before admission to the ICU might mask the relationship between weight and mortality by increasing mortality risk in patients categorized as 'overweight' (when in reality these patients are merely overhydrated) [46]. Our use of recent weights obtained in a period of good health from infant health records and other sources is likely to represent the true nutritional status of the patient. Nevertheless, a prospective study including accurate weight measurement on admission to the ICU should be performed before weight centile can be considered for inclusion in PIM or PRISM scores. Similarly, we did not measure height centile in our patients. It is likely that some patients in our study had low or high weight centiles that were accompanied by a comparably low or high height centile; that is, they were simply small or tall rather than nutritionally deficient or overweight. However, the number of proportionally small or tall patients is unlikely to exceed the normal population frequency; that is, we would expect 3% of our patients to fall into the lowest and highest 3 centiles at either end of the spectrum, not the 21.5% and 5.7% we observed. An association was observed between low weight centiles and the presence of chromosomal or syndromal disorders or both; however, the mortality of patients with these disorders was not different from the mortality of the population as a whole, suggesting that while such disorders are often associated with low weight centile, the presence of these disorders is not responsible for the increased mortality risk.

## Conclusions

Admission weights at the extremes of the centile range (low and high) are associated with an increased risk of mortality in the PICU, and patients with weights at the extremes of the centile range appear to be numerically over-represented in the ICU, although for overweight patients this may reflect simply the increasing incidence of obesity in the community. Given that our data suggest that inclusion of weight centile has the potential to improve the accuracy of mortality prediction (particularly in populations in which malnutrition may be more prevalent), a multicenter prospective study of this variable should be undertaken.

## Key messages

• Weight centile is an independent risk factor for mortality in patients admitted to pediatric intensive care; the lowest mortality occurs in patients with weights at the 75th centiles, and mortality increases as patient weights move away from the 75th centile toward either end of the weight spectrum.

• Weight centile should be considered for inclusion as a variable in mortality prediction models.

## Abbreviations

BMI: body mass index; ICU: intensive care unit; NICU: neonatal intensive care unit; OR: odds ratio; PICU: pediatric intensive care unit; PIM-2: Pediatric Index of Mortality version 2; PRISM: Pediatric Risk of Mortality; ROC: receiver operating characteristic.

## Competing interests

The authors declare that they have no competing interests.

## Authors' contributions

AN conceived of the study, analyzed the data, and drafted the manuscript. JM, GW, JA, and HR contributed equally to refining the study design, data collection, and manuscript revisions. All authors read and approved the final manuscript.
